# Supplementary material for: Gene Regulatory Network Inference from Single-Cell Data Using Multivariate Information Measures
Source: Cell Syst. 2017 Sep 27;5(3):251–267.e3. doi: 10.1016/j.cels.2017.08.014 (PMC5624513; doi:10.1016/j.cels.2017.08.014)
Supplement: Document S1. Figures S1–S4 and Tables S1–S3 [file mmc1.pdf]

Cell Systems, Volume *5*

Supplemental Information

Gene Regulatory Network Inference from Single-Cell Data Using Multivariate Information Measures

Thalia E. Chan, Michael P.H. Stumpf, and Ann C. Babtie

Supplementary Information for
Gene regulatory network inference from single-cell data using multivariate information measures

Thalia E Chan, Michael P H Stumpf, Ann C Babtie

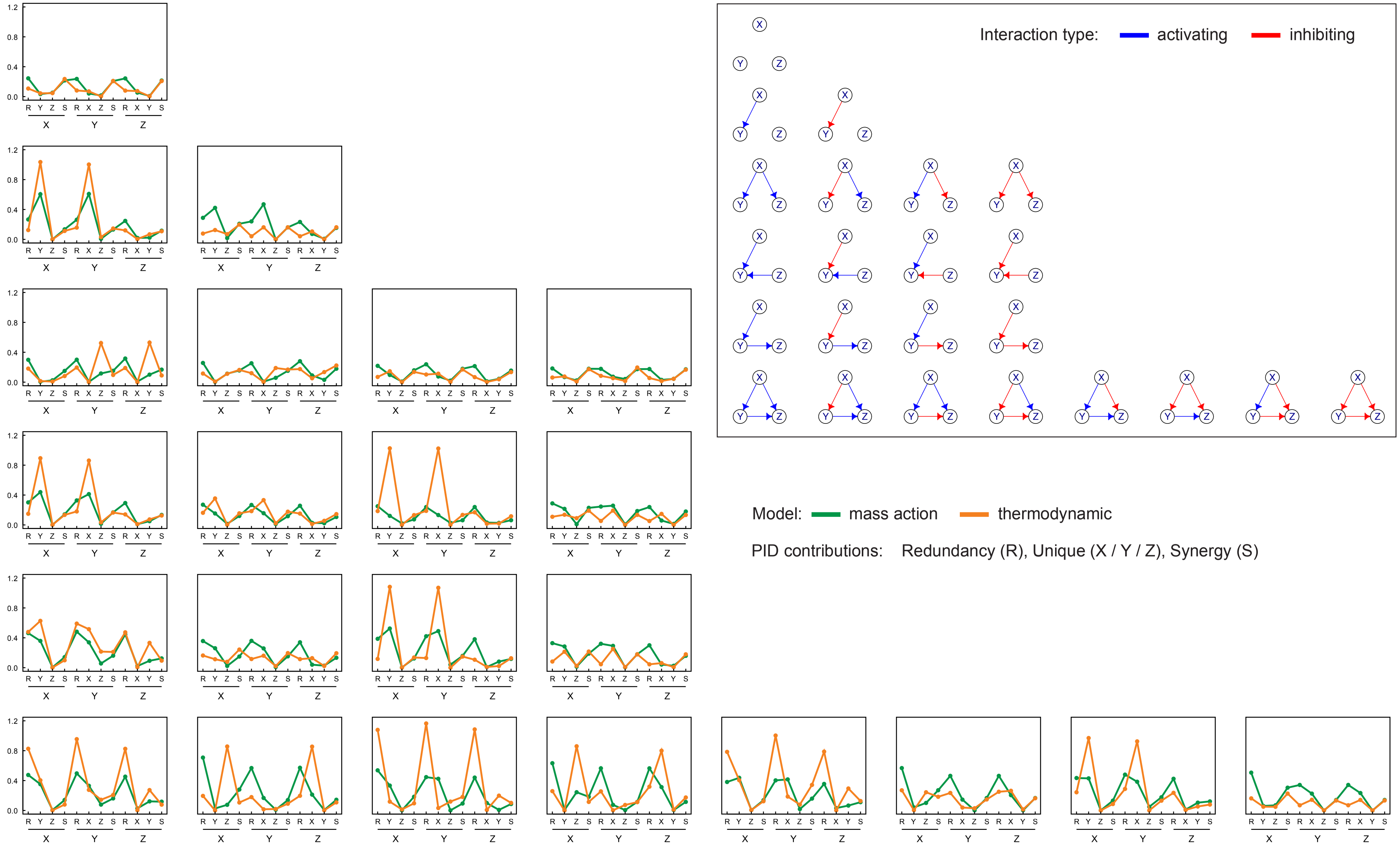

Figure S1: Related to Figure 2. Mean PID profiles for 3-gene network simulations, with stimulating ligand targeting gene X, and different network topologies. PID values were calculated using data simulated from 3-gene networks with the topologies illustrated in the top right inset (blue and red arrows indicate activating and inhibiting interactions respectively). Each line graph in the main figure shows the mean PID values calculated using models with the topology indicated in the equivalent grid position (i.e. the same row/column); the horizontal axis labels indicate the PID contribution, e.g. the first four values show the PID values with gene X as the target, consisting of the redundancy (R), unique contributions from gene Y (Y) and gene Z (Z), and the synergistic contribution (S). The models used for simulation assumed mass action (green) or thermodynamic (orange) kinetics, and the additional stimulation ligand targeted gene X from halfway through the simulation time (see *Methods*). The values plotted are the mean PID values calculated from five sets of simulations (with different randomly sampled initial conditions). Note that the same profiles are not necessarily seen for networks with equivalent connectivity, but different types of edges (results in the same row of this figure) — this is to be expected, as statistical relationships between genes can only be detected when there is sufficient variability in the observed data (which may not occur in some cases, e.g. if expression of a gene is inhibited throughout the simulation time).

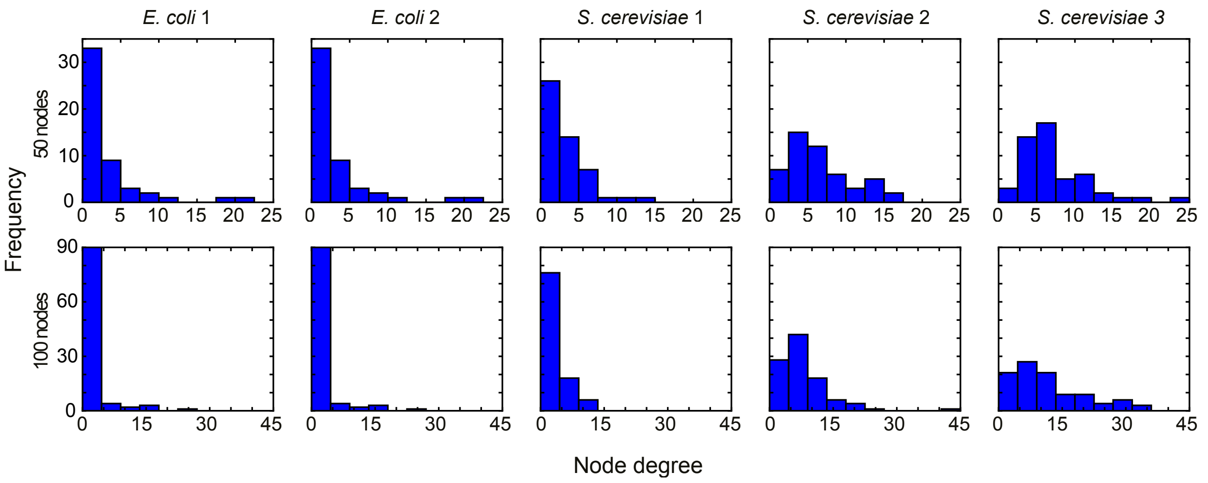

Figure S2: Related to Figure 2 and Table S1. Node degree distributions for each of the 50-node and 100-node networks produced by GeneNetWeaver (Schaffter et al. 2011). The distributions are varied, with the *E. coli* networks tending to have more hubs, and the *S. cerevisiae* networks tending to be more densely connected.

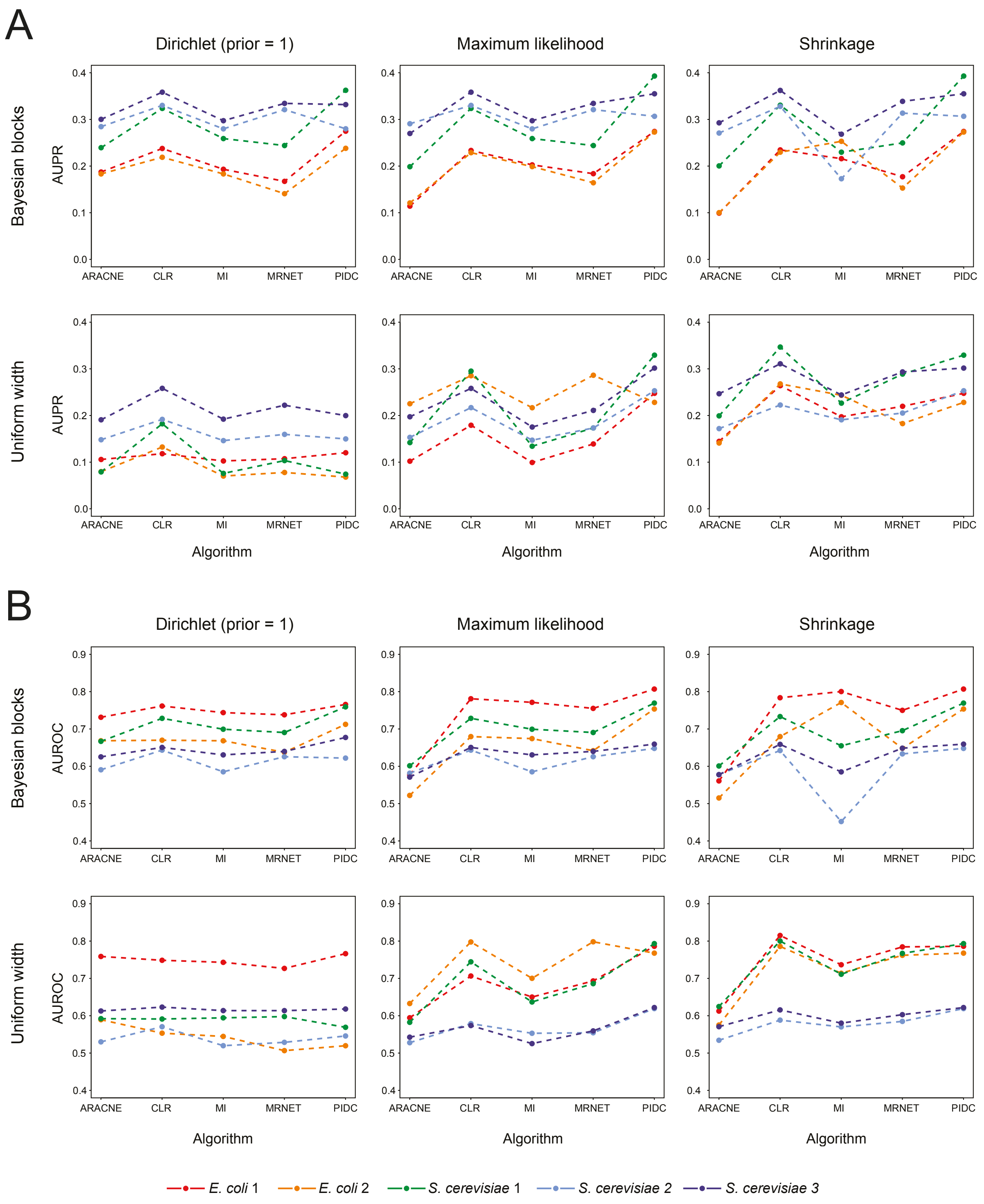

Figure S3: Related to Figure 5. Influence of discretization algorithm and estimator on network inference performance. AUPR (**A**) and AUROC (**B**) scores quantifying the accuracy of inferred networks calculated using *in silico* data simulated from five 50-gene networks (see *Methods*). Coloured lines indicate the results obtained with different datasets. Each plot shows the results obtained using a different combination of discretization algorithm (rows) and MI estimator (columns). Choice of algorithm and estimator clearly affects the relative scores of the network inference algorithms, suggesting that comparisons made using inconsistent combinations should be interpreted with caution; we find that the PIDC inference algorithm performs consistently well across the different combinations. The R package `minet` was used to implement the existing algorithms using the default parameters (Meyer et al. 2008).

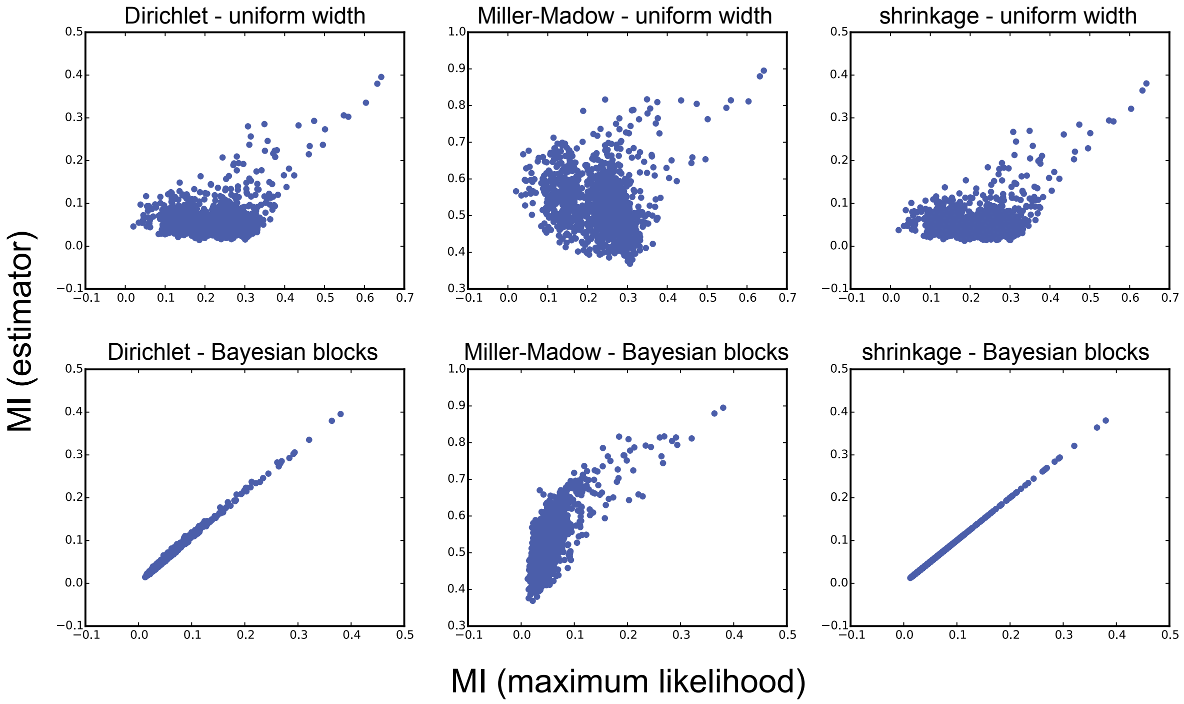

Figure S4: Related to Figure 5. Influence of discretization algorithm and estimator on MI rank. MI was estimated for every pair of genes in a 50-gene *in silico* network, using different combinations of discretization algorithms (uniform width or Bayesian blocks (Vanderplas et al. 2012, Scargle et al. 2013)) and MI estimators (Dirichlet, Miller-Madow, shrinkage and maximum likelihood); see *Methods* for details. Each plot shows the relative ranks of MI scores obtained using the maximum likelihood estimator (horizontal axis) versus one of the other estimators (vertical axis); the top and bottom rows show results obtained using data discretized using a uniform width or Bayesian blocks algorithm respectively. MI ranks were the most consistent when using Bayesian blocks discretization (with the exception of the Miller-Madow estimator, which is an entropy bias correction that should not be used in higher-dimensional estimation and is included here due to its frequent misuse as an MI estimator).

Table S1: Related to Figures 2 and S2. Topological characteristics of the 50-node and 100-node (in brackets) networks from which data were simulated by GeneNetWeaver (Schaffter et al. 2011). In all networks the vast majority of all possible node triples were either unconnected or had only one edge connecting two nodes in the triple.

Network	Nodes	Triples	Edges	Unconnected triples	One edge triples
E. coli 1	50 (100)	19600 (161700)	62 (125)	86.4% (92.8%)	12.0% (6.8%)
E. coli 2	50 (100)	19600 (161700)	82 (119)	82.8% (93.2%)	14.4% (6.3%)
S. cerevisiae 1	50 (100)	19600 (161700)	77 (166)	82.7% (90.3%)	15.9% (9.3%)
S. cerevisiae 2	50 (100)	19600 (161700)	160 (389)	66.6% (78.8%)	28.2% (18.9%)
S. cerevisiae 3	50 (100)	19600 (161700)	173 (551)	64.4% (71.6%)	29.7% (24.1%)

Table S2: Related to Figure S3 and Table S3. Joint entropy estimates, in bits, for up to four independent random variables, calculated using different estimators and uniform width discretization. Each variable is uniformly distributed over $n = 64$ bins and sampled 64^2 times, with theoretical entropy, $\log_2(n)$. The theoretical joint entropy of independent variables is the sum of their entropies. The Dirichlet estimator is given priors of $1/n$ or 1, and the shrinkage estimator is given a uniform target (as indicated in parentheses in table headings). The means of 100 repetitions are given, and the variances are all $< 10^{-4}$. Only the Dirichlet and shrinkage estimators produce accurate estimates with three and four variables.

Number of variables	Theoretical value	Maximum likelihood	Miller-Madow	Dirichlet ($1/n$)	Dirichlet (1)	Shrinkage (uniform)
1	6	5.9886	5.9963	5.9892	5.9998	6.0000
2	12	11.1705	11.4860	11.2234	11.8303	11.9998
3	18	11.9844	12.4804	15.9346	17.9915	17.9999
4	24	11.9997	12.4804	23.9283	23.9999	23.9998

Table S3: Related to Figure S3 and Table S2. Estimates of the difference between joint and marginal entropies of four independent random variables. The theoretical difference for independent variables is 0. Estimates are made for three sets of variables, drawn from three different distributions (uniform, normal or exponential). The Dirichlet estimator is given priors of $1/n$ and 1, and the shrinkage estimator is given a uniform target (as indicated in parentheses in table headings). The means of 100 repetitions are given, and the variances are all < 0.2. The estimators perform differently depending on the distribution: the Dirichlet and shrinkage estimator are the most accurate when given the correct prior, but can be the least accurate when the prior is wrong.

Distribution	Maximum likelihood	Miller-Madow	Dirichlet ($1/n$)	Dirichlet (1)	Shrinkage (uniform)
Uniform	11.9559	11.4868	0.0277	0.0428	0.0005
Normal	8.7172	8.2470	3.2126	3.1409	3.1389
Exponential	5.2646	4.8102	6.7636	6.5397	6.1207

References

Meyer, P.E., Lafitte, F., & Bontempi, G. (2008). minet: A R/Bioconductor Package for Inferring Large Transcriptional Networks Using Mutual Information. *BMC Bioinformatics*, 9(1):461–10.

Scargle, J.D., Norris, J.P., Jackson, B., & Chiang, J. (2013). Studies in Astronomical Time Series Analysis. VI. Bayesian Block Representations. *Astrophysical Journal*, 764:167. doi: 10.1088/0004-637X/764/2/167.

Schaffter, T., Marbach, D., & Floreano, D. (2011). GeneNetWeaver: in silico benchmark generation and performance profiling of network inference methods. *Bioinformatics*, 27(16):2263–2270.

Vanderplas, J., Connolly, A., Ivezić, Ž., & Gray, A. (2012). Introduction to astroML: Machine learning for astrophysics. In *Conference on Intelligent Data Understanding (CIDU)*, pages 47 –54. doi:10.1109/CIDU.2012.6382200.
